# Samarium-Doped Lead Phosphate Glass: Optical Experiments and Calculations Using the Judd–Ofelt Theory

**DOI:** 10.3390/ma18225254

**Published:** 2025-11-20

**Authors:** Joanna Pisarska, Wojciech A. Pisarski

**Affiliations:** Institute of Chemistry, University of Silesia, Szkolna 9 Street, 40-007 Katowice, Poland; wojciech.pisarski@us.edu.pl

**Keywords:** lead phosphate glass, Sm^3+^ ions, absorption, emission, Judd–Ofelt analysis

## Abstract

In this work, Sm^3+^-activated lead phosphate glass has been studied using spectroscopic methods. Based on absorption spectrum measurements, the oscillator strengths for Sm^3+^ ions were determined and compared to those calculated from the Judd–Ofelt theory. This procedure was applied to evaluate some radiative parameters (radiative transition rates, emission branching ratios, radiative lifetime) of Sm^3+^ ions in lead phosphate glass. Further luminescent studies indicate that lead phosphate glass doped with Sm^3+^ emits intense reddish-orange light due to ^4^G_5/2_ ⟶ ^6^H_7/2_ transition, for which several important spectroscopic parameters like emission linewidth and lifetime, quantum efficiency, peak stimulated emission cross-section, and figure of merit for laser gain were determined. The factors for Sm^3+^ ions in lead phosphate glass are as follows: η = 53%, FWHM = 10.5 nm, τ_exp_ = 1.925 ms, σ_em_ = 7.6 × 10^−22^ cm^2^, σ_em_ × τ_exp_ = 14.6 × 10^−25^ cm^2^s. The experimental and theoretical results suggest that samarium-doped lead phosphate glass can be successfully used as a reddish-orange-emitting component in photonic devices.

## 1. Introduction

Samarium-doped inorganic glasses, due to their enhanced luminescent transition ^4^G_5/2_ ⟶ ^6^H_7/2_ (Sm^3+^), are known as promising reddish-orange emitting materials useful in solid-state lighting (SSL) technology and photonic devices [1,2,3,4,5]. Emission properties have been examined for numerous glass systems doped with Sm^3+^ ions, i.e., silicate [6,7,8,9,10], germanate [11,12,13], tellurite [14,15,16], and multicomponent mixed network-former glasses [17,18,19,20,21,22,23,24,25,26,27]. Luminescence behavior of Sm^3+^ ions in non-oxide glasses (chalcogenide, fluoride) has also been explored [28,29,30,31]. A great attention has been paid to borate [32,33,34,35,36,37,38,39,40] and phosphate [41,42,43,44,45,46,47,48,49] glasses containing Sm^3+^ ions. The impact of fluoride and oxide network modifiers on local structure and physicochemical properties (especially luminescence) of Sm^3+^-doped glasses has been analyzed in detail [50,51,52,53]. From numerous literature data, it is also well-known that trivalent samarium ions play an important role as an excellent acceptor in Dy^3+^/Sm^3+^ [54,55,56,57,58,59] and Tb^3+^/Sm^3+^ [60,61,62,63,64,65] co-doped glass systems through the excitation energy transfer processes.

Among inorganic glass systems, rare-earth-activated lead phosphate glasses represent a family of heavy metal glass (HMG) systems [66,67,68,69,70,71,72], and they are really attractive for optical applications. Recent studies concerned with rare-earth-activated lead phosphate-based glasses emitting visible light [73,74,75,76] and near-infrared radiation [76,77,78,79]. Owing to the hygroscopic nature of the main component P_2_O_5_, the procedure for glass synthesis should be strongly restrictive, i.e., glass samples should be synthesized in a glove-box in order to eliminate hydroxyl groups, which quench emission from excited states of rare earths [80]. Our previous investigations for rare-earth-activated lead phosphate glasses revealed that the intensities of the IR band assigned to the vibration of the hydroxyl groups were significantly lower for glasses fabricated in a glove box than in open air [81]. Thus, luminescence characteristics and spectroscopic parameters for rare earths are enhanced significantly, which is extremely important from the optical point of view.

In this work, lead phosphate glass based on PbO-P_2_O_5_-Ga_2_O_3_-Sm_2_O_3_ composition has been studied using optical spectroscopy. The introduction of the third component Ga_2_O_3_ to several glass host matrices increases their thermal stability [82]. It was also confirmed for lead phosphate glasses, for which the thermal stability increases with increasing Ga_2_O_3_ content [83]. The Sm^3+^ concentration was close to 0.5 mol%. From the luminescent studies published previously, low-concentrated (0.5 mol%) Sm^3+^ doped glasses [84,85,86,87,88,89] showed optical maximum emission intensity in the reddish-orange spectral region. Above 0.5 mol% of Sm^3+^, the activator concentration effects in glass samples are clearly observed due to cross-relaxation processes occurring between samarium ions [90,91,92]. Based on the optical experiments (absorption and emission spectra measurements) and calculations using the standard Judd–Ofelt theory [93,94], several spectroscopic and laser parameters of samarium ions in lead phosphate glass were determined. At this moment, it should be pointed out that the Judd–Ofelt calculation strategy was greatly developed, and some calculation routes were established in recent years. The Judd–Ofelt parameters of rare earths have been determined according to the excitation spectrum [95]. Luo et al. [96] have established a fluorescence decay route to calculate the Judd–Ofelt parameters of rare earth ions. In the current work, measured and calculated spectroscopic parameters were compared to the previous results published for similar Sm^3+^-doped glasses. The optical results indicate that Sm^3+^-activated lead phosphate glass is promising for reddish-orange emission applications.

## 2. Materials and Methods

Lead phosphate glass with the following chemical formula given in mole percent (mol%) 45PbO-45P_2_O_5_-9.5Ga_2_O_3_-0.5Sm_2_O_3_ was prepared by mixing and melting amounts of P_2_O_5_ and appropriate metal oxides. Oxide components of high purity (99.99%, Aldrich Chemical Co., St. Louis, MO, USA) were used to prepare a glass sample in a glove box, where a homogeneous mixture was heated in a protective atmosphere of dried argon (99.99%). In our case, the argon is necessary, contrary to the cheaper, but strongly reducing atmosphere of nitrogen, which can effectively reduce Sm^3+^ to Sm^2+^ ions. Reagents were melted in a Pt crucible at T = 1100 °C for 0.5 h.

In order to characterize the prepared glass, the XRD pattern (X’Pert Pro diffractometer, Panalytical, Almelo, The Netherlands), the DSC curve (Perkin Elmer differential scanning calorimeter, Shelton, CT, USA), and the Raman spectrum (Thermo Scientific^TM^ DXR^TM^2xi Raman imaging microscope, Waltham, MA, USA) were measured, whereas the Metricon 2010 prism coupler (Pennington, NJ, USA) using λ = 632.8 nm was used to estimate the refractive index. Absorption and emission spectra were measured using a UV-VIS-NIR spectrophotometer (Cary 5000, Agilent Technology, Santa Clara, CA, USA) and a PTI QM40 spectrofluorometer (Photon Technology International, Birmingham, NJ, USA). Details for the equipment of the spectrofluorometer are given in previous work [97]. Resolution for the absorption and emission spectra was ±0.1 nm. The decay emission curve was measured with an accuracy of ±1 μs. All measurements were carried out at room temperature.

## 3. Results

**Figure 1:**
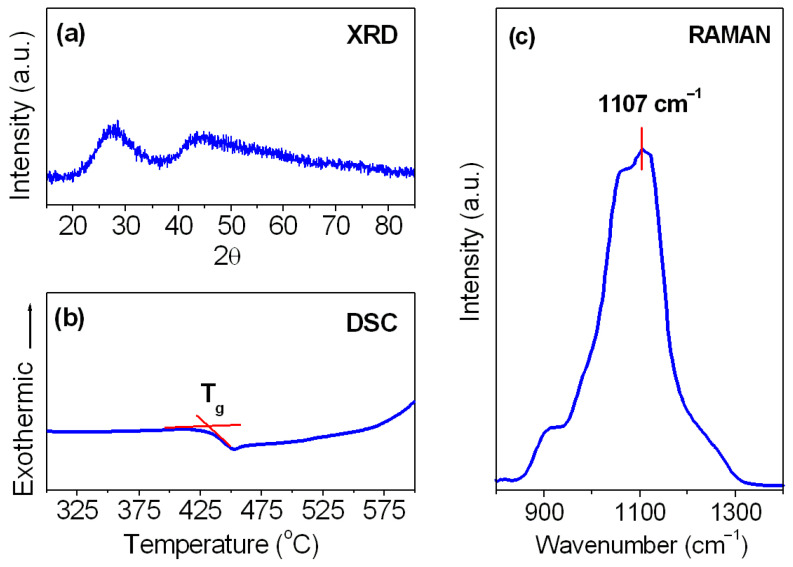
(**a**) XRD pattern, (**b**) DSC curve and (**c**) the Raman spectrum for Sm^3+^-activated lead phosphate glass.

Figure 1 presents the XRD pattern, DSC curve and Raman spectrum for Sm^3+^-activated lead phosphate glass. The X-ray diffraction analysis indicated that the studied sample is fully amorphous without narrow diffraction lines characteristic of crystalline systems. However, we do not exclude the presence of several small crystalline particles inside the glass that cannot be detected due to the resolution limit. The DSC curve was registered for the glass sample up to 600 °C, because the operating temperature range of the calorimeter is limited. Based on the DSC curve measurement, the glass transition temperature T_g_ was estimated, and its value is equal to 435 °C. Owing to the lack of the exothermic peak in the temperature range between T_g_ and T = 600 °C, we suggest that Sm^3+^-activated lead phosphate glass exhibits relatively good thermal stability (ΔT larger than 165 °C) against devitrification. The Raman spectrum shows that several vibration modes related to the characteristic phosphate groups are involved in the studied glass. The assignments of the unresolved bands for lead fluorophosphate glass were reported earlier by Kesavulu and Jayasankar [98]. The Raman bands centered at about 1050 cm^−1^ and 1107 cm^−1^ are due to symmetric stretching vibration of diphosphate and metaphosphate groups, whereas the shoulders near 900 cm^−1^ and 1250 cm^−1^ correspond to the symmetric (PO_4_ groups) and asymmetric (PO_2_ groups) stretching vibrations, respectively. Additionally, the broad Raman band located at about 750 cm^−1^ (not presented here) is assigned to P–O–P stretching vibration [98]. Phonon energy of the studied glass (mode with maximum energy) obtained from the Raman spectrum measurements is equal to nearly 1107 cm^−1^. For that reason, it was suggested that lead phosphate glass belongs rather to a medium-phonon system located between high-phonon borate-based glasses (hω = 1300 ÷ 1400 cm^−1^) and low-phonon tellurite or germanate glass matrices (700 ÷ 800 cm^−1^), respectively.

The Raman results are well correlated with the following value 1117 cm^−1^, which was obtained for Eu^3+^-activated lead phosphate glass from the phonon sideband excitation spectrum measurements [99]. Selected physicochemical properties of Sm^3+^-activated lead phosphate glass are summarized in Table 1.

**Figure 2:**
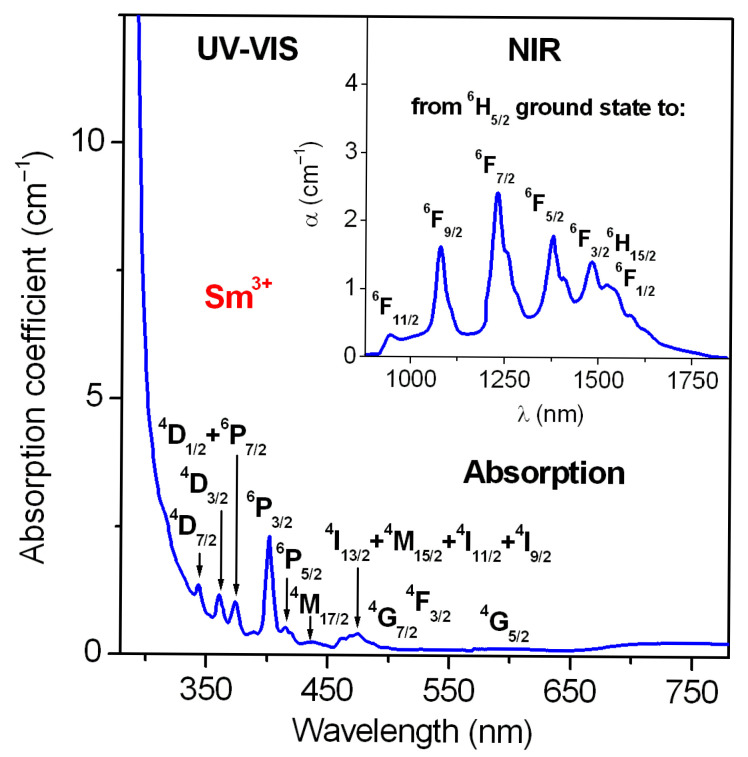
UV-VIS absorption spectrum of Sm^3+^-activated lead phosphate glass. Inset presents the absorption measured in the NIR spectral range.

In the next step, the absorption spectrum of Sm^3+^-doped lead phosphate glass was measured in the UV-VIS and NIR spectral range. The cut-off wavelength is close to 304 nm (UV range). The spectrum consists of bands characteristic of the 4f^5^-4f^5^ electronic intraconfigurational transitions of samarium ions. Absorption bands are assigned to transitions originating from ^6^H_5/2_ ground state to the following excited states of Sm^3+^: ^4^D_7/2_, ^4^D_3/2_, ^4^D_1/2_ + ^6^P_7/2_, ^6^P_3/2_, ^6^P_5/2_, ^4^M_17/2_, ^4^M_15/2_ + ^4^I_J/2_ (J = 9, 11, 13, 15), ^4^G_7/2_, ^4^F_3/2_, ^4^G_5/2_ (UV-VIS absorption range) and ^6^F_11/2_, ^6^F_9/2_, ^6^F_7/2_, ^6^F_5/2_, ^6^F_3/2_, ^6^H_15/2_ and ^6^F_1/2_ (NIR absorption range), respectively. The absorption spectrum for Sm^3+^-doped lead phosphate glass is presented in Figure 2. Then, the standard Judd–Ofelt framework [93,94] was used to calculate the theoretical oscillator strengths for each transition of Sm^3+^ ions in lead phosphate glass, which were compared to the measured oscillator strengths estimated by measuring the areas under the absorption bands (Figure 2). The absorption bands from the ^6^H_5/2_ ground state to the ^6^F_J_ excited states with the spin selection rule ΔS = 0 were taken into account in the Judd–Ofelt calculation, because these transitions are allowed and their intensities are relatively strong [20]. The absorption transitions in the UV-VIS region are overlapped, and the energy states are lying very close to each other, thus the Judd–Ofelt calculations become more complicated. The calculation procedure using the Judd–Ofelt theory has been successfully applied to numerous Sm^3+^-activated inorganic glass systems [100,101,102,103,104,105,106,107].

The experimental and theoretical oscillator strengths of Sm^3+^ ions were determined using the following relations:

(1) $$P_{\mathrm{meas}}=4.318\times 10^{-9}\int \varepsilon (\nu )d\nu$$

where ∫ε(ν)dv is the area under the absorption band and ε(ν) = A/c × l. In this relation, A, c and l denote the absorbance, the Sm^3+^ concentration and the optical path length.

(2) $$P_{\mathrm{calc}}=\frac{8\pi^{2}\mathrm{mc}{\left(n^{2}+2\right)}^{2}}{3h\lambda (2J+1)\cdot 9n}\times \sum_{t=2,4,6}\Omega_{t}{\left(<4f^{N}J\|U^{t}\|4f^{N}J^{\prime }>\right)}^{2}$$

where m, c, h and λ denote the electron mass, the light velocity, the Planck constant and the mean wavelength of each transition. Here, ║U^t^║^2^ for samarium ions was adopted from Ref. [108]. The experimental and theoretical oscillator strengths for samarium ions in lead phosphate glass are given in Table 2. Small deviation of root mean square value (rms = ±0.34 × 10^−6^) between P_meas_ and P_calc_ suggests good fitting between them.

Based on comparison of the experimental and theoretical oscillator strengths for Sm^3+^ ions, the Judd–Ofelt intensity parameters Ω_t_ (where t = 2, 4, 6) were determined and compared to some glass systems [109,110,111,112]. The results are shown in Table 3.

In the following step, the factors Ω_t_ were applied to obtain the radiative transition rates and emission branching ratios using the following relation:

(3) $$A_{J}=\frac{64\pi^{4}e^{2}}{3h(2J+1)\lambda^{3}}\times \frac{n{\left(n^{2}+2\right)}^{2}}{9}\times \sum_{t=2,4,6}\Omega_{t}{\left(<4f^{N}J\|U^{t}\|4f^{N}J^{\prime }>\right)}^{2}$$

(4) $$\beta =\frac{A_{J}}{\sum_{i}A_{Ji}}$$

The radiative transition rates and emission branching ratios for samarium in lead phosphate glass are presented in Table 4. The total radiative transition rate, defined as the sum of the A_J_ values calculated for each transition from the ^4^G_5/2_ excited state to the lower lying states of Sm^3+^ ions, is equal to A_TOTAL_ = 275.5 s^−1^, whereas the radiative lifetime τ_rad_ for the state ^4^G_5/2_ (Sm^3+^) as an inverse of A_TOTAL_ is close to 3.63 ms. Details for the Judd–Ofelt analysis and calculations using relations (1)–(4) are given elsewhere [97].

**Figure 3:**
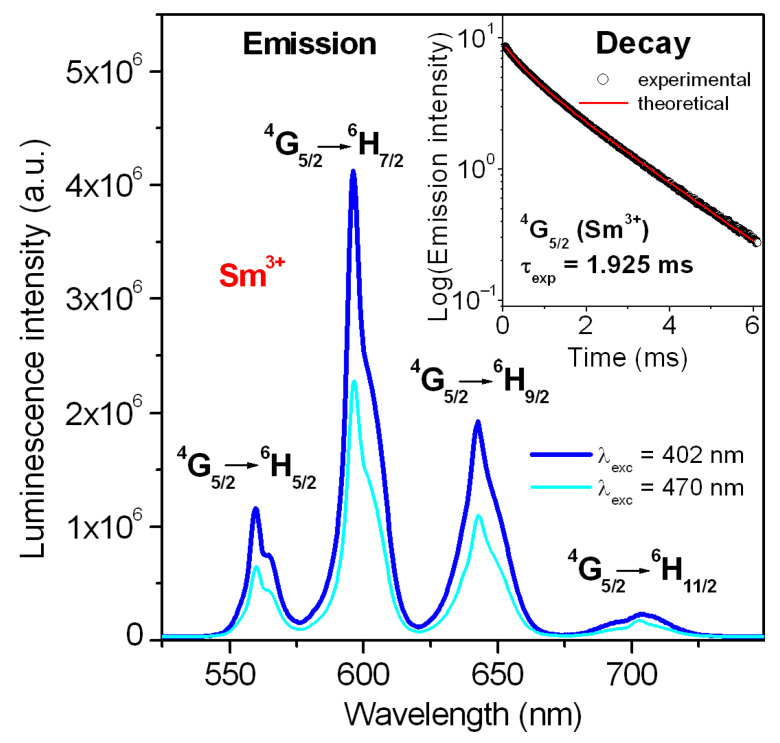
Emission spectrum of Sm^3+^-activated lead phosphate glass. Inset presents emission decay curve for the ^4^G_5/2_ excited state of Sm^3+^.

In the next step, emission properties have been examined in detail. The emission spectrum of Sm^3+^-activated lead phosphate glass is presented in Figure 3.

**Figure 4:**
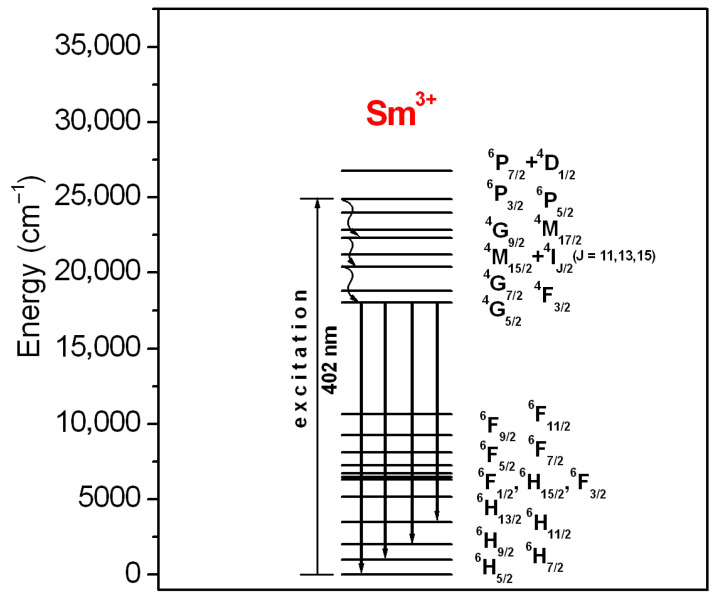
Energetic diagram for samarium-activated lead phosphate glass. Luminescent transitions of Sm^3+^ ions are also indicated.

The results well demonstrated that the emission intensity is higher for the glass sample excited at 402 nm (^6^P_3/2_ state) than 470 nm (multiband due to ^4^M_15/2_ + ^4^I_J/2_ (J = 9, 11, 13, 15) states), respectively. Four emission bands correspond to ^4^G_5/2_ ⟶ ^6^H_J/2_ (where J = 5, 7 9, 11) transitions of samarium. Reddish-orange emission at 596 nm due to the ^4^G_5/2_ ⟶ ^6^H_7/2_ transition of samarium ions is the most intense. The inset shows a decay curve for the ^4^G_5/2_ state of samarium ions in lead phosphate glass. Luminescence lifetime was estimated based on the decay curve measurement. Its experimental value τ_exp_ for the excited state ^4^G_5/2_ (Sm^3+^) is close to 1.925 ms. Four emission lines are illustrated schematically on the energy level diagram of Sm^3+^ ions in Figure 4.

The experimental emission lifetime τ_exp_ as well as the radiative lifetime τ_rad_ and the radiative transition rate A_J_ from the Judd–Ofelt framework were used to calculate the quantum efficiency η and the peak stimulated emission cross-section σ_em_. The appropriate relations (5) and (6) are given below:

(5) $$\eta =\frac{\tau_{\exp}}{\tau_{\mathrm{rad}}}\times 100\%$$

(6) $$\sigma_{\mathrm{em}}=\frac{\lambda_{p}^{4}}{8\pi cn^{2}\Delta \lambda }A_{J}$$

In relation (6), n is the refractive index, and its value is close to 1.75 (Table 1), λ_p_ is the peak emission wavelength, whereas Δλ is the effective linewidth defined as full width at half maximum (FWHM).

Spectroscopic parameters for samarium ions in lead phosphate glass are given in Table 5. The results are compared to the previously published data for similar phosphate-based glass systems and heavy metal glasses containing lead [113,114,115,116,117,118,119,120,121,122,123].

## 4. Discussion

The physicochemical results obtained using XRD, DSC and Raman methods (Figure 1) confirmed that Sm^3+^-doped lead phosphate glass is fully amorphous, thermally stable and belongs to medium-phonon glass matrices (Table 1). Based on the absorption spectrum recorded in the UV-VIS and NIR ranges (Figure 2) and relations (1)–(4) from the Judd–Ofelt theory, several radiative parameters for samarium ions in lead phosphate glass were obtained. The measured and calculated oscillator strengths for transitions of Sm^3+^ ions were compared (Table 2), and the Judd–Ofelt parameters Ω_t_ (t = 2, 4, 6) were achieved. The following trend Ω_4_ > Ω_6_ > Ω_2_ or Ω_6_ > Ω_4_ > Ω_2_ is usually observed for Sm^3+^ ions in inorganic glasses [124]. In our case, the Judd–Ofelt intensity parameters for Sm^3+^ ions are changed in direction Ω_6_ > Ω_4_ > Ω_2,_ and this trend is similar to the results obtained earlier for other systems (Table 3). Moreover, the Judd–Ofelt intensity parameter Ω_2,_ exhibiting the degree of bonding (covalent/ionic) between samarium ions and ligands is low. Its value is equal to 0.76 × 10^−20^ cm^2^. It suggests absolutely more ionic bonding in character between samarium ions and their nearest surroundings confirming previous Judd–Ofelt results obtained for phosphate-based glasses doped with Sm^3+^ [125,126,127].

The Judd–Ofelt parameters Ω_t_ were used to obtain the radiative transition rates, emission branching ratios and radiative lifetime (Table 4). In particular, the luminescence properties of Sm^3+^-doped lead phosphate glass have been examined in detail. Luminescence spectrum (Figure 3) consists of four bands, which are associated with the following transitions: ^4^G_5/2_ ⟶ ^6^H_5/2_, ^4^G_5/2_ ⟶ ^6^H_7/2_, ^4^G_5/2_ ⟶ ^6^H_9/2_ and ^4^G_5/2_ ⟶ ^6^H_11/2_ centered at 560 nm, 596 nm, 648 nm and 710 nm, respectively. All transitions are indicated on the energetic diagram of samarium ions (Figure 4). Lead phosphate glass with Sm^3+^ emits reddish-orange light related to the most intense ^4^G_5/2_ ⟶ ^6^H_7/2_ transition at 596 nm. Based on emission spectrum and its decay from the ^4^G_5/2_ (Sm^3+^), and the appropriate relations (5) and (6), several spectroscopic parameters for trivalent samarium ions in lead phosphate glass were determined. The results are summarized in Table 5.

The main spectroscopic parameters for Sm^3+^ ions in PbO-P_2_O_5_-Ga_2_O_3_ glass system are as follows: the peak emission wavelength λ_p_ = 596 nm, the luminescence linewidth FWHM = 10.5 nm, the ^4^G_5/2_ theoretical radiative lifetime τ_rad_ = 3.63 ms, the experimental luminescence lifetime of ^4^G_5/2_ state τ_exp_ = 1.925 ms, the stimulated emission cross-section σ_em_ = 7.6 × 10^−22^ cm^2^ and the quantum efficiency ^4^G_5/2_ (Sm^3+^) η = 53%. The latter parameter, i.e., the quantum efficiency η is larger than 50% suggesting that Sm^3+^-activated lead phosphate glass is also a potential laser material emitting in the reddish-orange region. Interestingly, the quantum efficiency for the ^4^G_5/2_ (Sm^3+^) state in lead phosphate glass is smaller than the η value (60.4%) obtained for Sm^3+^ ions in heavy metal oxide glass based on Li_2_O-PbO-Al_2_O_3_-B_2_O_3_ [113] but higher than η = 45.6% for Sm^3+^ in heavy metal oxyfluoride glass based on PbF_2_-TeO_2_-WO_3_ [114]. In fact, the quantum efficiency agrees with the results (54–58%) for multicomponent heavy metal oxide glass [115] and phosphate-based glasses with the presence [116] and absence [117,118] of lead. Contrary to η values, the peak stimulated emission cross-section σ_em_ for the main ^4^G_5/2_ ⟶ ^6^H_7/2_ reddish-orange transition of trivalent samarium ions in lead phosphate glass is higher compared to the values (σ_em_ = 5.8 ÷ 7.23 × 10^−22^ cm^2^) obtained for heavy metal glass systems as well as phosphate-based glasses [113,114,115,116,117,118]. At this moment, it should also be noted that both spectroscopic parameters seem to be smaller compared to the values η (77 ÷ 98%) and σ_em_ (9 ÷ 12.4 × 10^−22^ cm^2^) reported for some phosphate-based glasses with Sm^3+^ [119,120,121,122,123].

Finally, the figure of merit for laser gain defined as σ_em_ × τ_exp_ was determined for the ^4^G_5/2_ ⟶ ^6^H_7/2_ transition of Sm^3+^ ions in lead phosphate glass at 596 nm. Relatively large values of σ_em_ × τ_exp_ product are necessary to generate laser action in glass systems. In our case, the figure of merit for laser gain is close to 14.6 × 10^−25^ cm^2^s and its value is higher compared to the following results: 8.51 × 10^−25^ cm^2^s for PbF_2_-TeO_2_-WO_3_ [114], 10.4 × 10^−25^ cm^2^s for P_2_O_5_-K_2_O-MgO-Al_2_O_3_ [118], 11.7 × 10^−25^ cm^2^s for Li_2_O-PbO-Al_2_O_3_-B_2_O_3_ [113], as well as 12.8 × 10^−25^ cm^2^s for P_2_O_5_-PbO-Nb_2_O_5_ [116], respectively. The σ_em_ × τ_exp_ product for trivalent samarium ions in lead phosphate glass ranges between 13.54 × 10^−25^ cm^2^s for P_2_O_5_-K_2_O-Al_2_O_3_-Nb_2_O_5_ [122] and 16.1 × 10^−25^ cm^2^s for P_2_O_5_-K_2_O-Al_2_O_3_-PbF_2_-Na_2_O [119], even though these Sm^3+^-doped glass systems exhibit higher values of η and σ_em_ compared to our glass, as mentioned above. It suggests that the figure of merit for laser gain can be useful in comparison to reported laser phosphate glass systems with Sm^3+^ ions [119,120,121,122,123]. According to the previous results for PbO-P_2_O_5_-Ln_2_O_3_ (Ln = Sm, Gd) glass without Ga_2_O_3_, the lifetimes and the quantum efficiencies depend significantly on the Sm^3+^ content as well as on the heating time of glasses melted at high temperature [128]. On the other hand, thermal stability increases with increasing Ga_2_O_3_ concentration in lead phosphate glass, as mentioned in the Section 1 [83]. Therefore, we can conclude based on the spectroscopic results shown in Table 5 that the thermally stable samarium-doped lead phosphate glass modified by Ga_2_O_3_ is suitable for the applications of reddish-orange visible light. It can be successfully used as a glass component in photonic devices similar to other Sm^3+^-doped glass host matrices published recently, which seem to be excellent reddish-orange-emitting candidates for numerous luminescence applications [129]. For example, borosilicate glasses modified by alkaline/alkali (CaF_2_, NaF) fluorides [87] or mixed alkali (Li_2_O, Na_2_O) oxides [130] playing the role as the glass-network-modifiers are attractive for luminescence applications after Sm^3+^ ion doping. Based on the structural, thermal and optical studies, it was suggested that borosilicate-based glasses doped with Sm^3+^ ions are promising for radiation protection and photonic display devices [87] and luminescence applications in art and decoration [130].

## 5. Conclusions

Optical experiments and the Judd–Ofelt calculations have been successfully applied to Sm^3+^-doped lead phosphate glass, which emits intense reddish-orange light related to ^4^G_5/2_ ⟶ ^6^H_7/2_ transition at 596 nm. Several spectroscopic factors for trivalent Sm^3+^ were determined. The following spectroscopic parameters the for ^4^G_5/2_ ⟶ ^6^H_7/2_ transition of samarium in lead phosphate glass were achieved: the quantum efficiency η = 53%, the emission linewidth FWHM = 10.5 nm, the experimental emission lifetime τ_exp_ = 1.925 ms, the peak stimulated emission cross-section σ_em_ = 7.6 × 10^−22^ cm^2^ and the figure of merit for laser gain σ_em_ × τ_exp_ = 14.6 × 10^−25^ cm^2^s, respectively. The results compared to heavy metal glass systems and phosphate-based glasses published previously. The theoretical and experimental studies demonstrate that Sm^3+^-activated lead phosphate glass is a promising reddish-orange laser gain media operated at 596 nm.

## Figures and Tables

**Table 1:** Some physicochemical properties of Sm^3+^-activated lead phosphate glass.

Parameters	Lead Phosphate Glass
Chemical composition (molar%)	45PbO-45P_2_O_5_-9.5Ga_2_O_3_-0.5Sm_2_O_3_
Average molecular weight (M g mol^−1^)	183.9
Density (d g cm^−3^)	5.12
Activator (Sm^3+^) content (molar %)	0.5
Sm^3+^ ion concentration (N × 10^20^ ions cm^−3^)	1.68
Refractive index (n)	1.75
Phonon energy (hω cm^−1^)	1107
Glass transition temperature (T_g_ °C)	435

**Table 2:** Measured and calculated oscillator strengths (P × 10^−6^) for lead phosphate glass with Sm^3+^.

Level	Energy (cm^−1^)	P_meas_	P_calc_	P_meas_ − P_calc_ *
^6^F_1/2_	6300	0.257	0.262	0.005
^6^F_3/2_	6750	0.880	0.877	0.003
^6^F_5/2_	7257	1.510	1.528	0.018
^6^F_7/2_	8130	3.950	3.905	0.045
^6^F_9/2_	9276	2.850	2.916	0.066
^6^F_11/2_	10,600	0.495	0.484	0.011

* rms = (∑(P_meas_ − P_calc_)^2^/N)^1/2^ = ±0.34 × 10^−6^, N—the number of transitions.

**Table 3:** Judd–Ofelt parameters for Sm^3+^ in lead phosphate glass compared to some glass systems.

Glass Host	Judd–Ofelt Parameters Ω_t_ (in 10^−20^ cm^2^ Units)			Trend	Ref.
	Ω_2_	Ω_4_	Ω_6_		
45PbO-45P_2_O_5_-9.5Ga_2_O_3_-0.5Sm_2_O_3_	0.76 ± 0.19	2.43 ± 0.09	2.98 ± 0.05	Ω_2_ < Ω_4_ < Ω_6_	this work
68H_3_BO_3_-30PbO-2Sm_2_O_3_	0.75	2.67	3.01	Ω_2_ < Ω_4_ < Ω_6_	[109]
67H_3_BO_3_-12Li_2_CO_3_-20Cs_2_CO_3_-1Sm_2_O_3_	0.81	3.22	4.28	Ω_2_ < Ω_4_ < Ω_6_	[110]
67B_2_O_3_-12Li_2_O-20K_2_O-1Sm_2_O_3_	0.88	3.93	4.66	Ω_2_ < Ω_4_ < Ω_6_	[111]
67B_2_O_3_-12Li_2_O-20Na_2_O-1Sm_2_O_3_	0.77	6.39	7.09	Ω_2_ < Ω_4_ < Ω_6_	[111]
67B_2_O_3_-12Na_2_O-20K_2_O-1Sm_2_O_3_	1.47	2.00	3.09	Ω_2_ < Ω_4_ < Ω_6_	[111]
30PbF_2_-30TeO_2_-39H_3_BO_3_-1Sm_2_O_3_	0.21	1.42	1.8	Ω_2_ < Ω_4_ < Ω_6_	[112]

**Table 4:** Radiative transition rates and emission branching ratios for Sm^3+^-activated lead phosphate glass.

Transition	λ (nm)	A_J_ (s^−1^) *	β
^4^G_5/2_ ⟶ ^6^F_11/2_	1460	0.34	0.11
^4^G_5/2_ ⟶ ^6^F_19/2_	1198	0.96	0.35
^4^G_5/2_ ⟶ ^6^F_7/2_	1038	2.60	0.94
^4^G_5/2_ ⟶ ^6^F_5/2_	953	6.82	2.48
^4^G_5/2_ ⟶ ^6^F_3/2_	908	0.82	0.30
^4^G_5/2_ ⟶ ^6^H_15/2_	902	0.50	0.18
^4^G_5/2_ ⟶ ^6^F_1/2_	893	0.66	0.24
^4^G_5/2_ ⟶ ^6^H_13/2_	795	7.16	2.60
^4^G_5/2_ ⟶ ^6^H_11/2_	710	32.87	11.93
^4^G_5/2_ ⟶ ^6^H_9/2_	648	70.47	25.58
^4^G_5/2_ ⟶ ^6^H_7/2_	596	145.84	52.94
^4^G_5/2_ ⟶ ^6^H_5/2_	560	6.46	2.35

* A_TOTAL_ = ∑A_J_ = 275.5 s^−1^, τ_rad_ = 1/A_TOTAL_ = 3.63 ms.

**Table 5:** Spectroscopic parameters for Sm^3+^ ions in lead phosphate glass and other glass systems.

Glass Composition (mol%)	λ_p_ (nm)	FWHM (nm)	τ_exp_ (ms)	τ_rad_ (ms)	η (%)	σ_em_ (cm^2^ × 10^−22^)	Ref.
45PbO-45P_2_O_5_-9.5Ga_2_O_3_-0.5Sm_2_O_3_	596	10.50	1.925	3.630	53	7.60	this work
10Li_2_O-10PbO-9Al_2_O_3_-70B_2_O_3_-1Sm_2_O_3_	597	10.59	0.966	1.600	60.4	7.23	[113]
15PbF_2_-59TeO_2_-25WO_3_-1Sm_2_O_3_	600	-	0.620	1.360	45.6	6.26	[114]
26.66B_2_O_3_-52.33PbO-16GeO_2_-4Bi_2_O_3_-1Sm_2_O_3_	601	15.03	1.392	2.580	54	6.08	[115]
55P_2_O_5_-39PbO-5Nb_2_O_5_-1Sm_2_O_3_	598	10.40	1.896	3.268	58	6.76	[116]
55P_2_O_5_-14K_2_O-6KF-15BaO-9Al_2_O_3_-1Sm_2_O_3_	597	12	2.400	4.290	56	5.92	[117]
58.5P_2_O_5_-15K_2_O-16.5MgO-9Al_2_O_3_-1Sm_2_O_3_	598	11.20	1.800	3.140	57	5.80	[118]
44P_2_O_5_-17K_2_O-9Al_2_O_3_-23PbF_2_-6Na_2_O-1Sm_2_O_3_	601	13.96	1.580	2.04	77	8.98	[119]
41P_2_O_5_-17K_2_O-8Al_2_O_3_-23ZnF_2_-10LiF-1Sm_2_O_3_	601	14.63	1.690	2.124	80	9.53	[120]
44P_2_O_5_-17K_2_O-9Al_2_O_3_-23PbO-6Na_2_O-1Sm_2_O_3_	598	11.20	1.570	1.880	83.5	11.50	[121]
44P_2_O_5_-17K_2_O-9Al_2_O_3_-29Nb_2_O_5_-1Sm_2_O_3_	602	15.07	1.175	-	98	11.52	[122]
45P_2_O_5_-45Na_2_O-2Al_2_O_3_-8BaO-0.5Sm_2_O_3_	600	-	2.400	2.710	88	12.36	[123]

## Data Availability

The original contributions presented in this study are included in the article. Further inquiries can be directed to the corresponding author.
